# Efficacy of Brazilian Propolis against Herpes Simplex Virus Type 1 Infection in Mice and Their Modes of Antiherpetic Efficacies

**DOI:** 10.1155/2011/976196

**Published:** 2011-06-14

**Authors:** Tomomi Shimizu, Youhei Takeshita, Yasushi Takamori, Hisahiro Kai, Rie Sawamura, Hiroki Yoshida, Wataru Watanabe, Atsuko Tsutsumi, Yong Kun Park, Ken Yasukawa, Koji Matsuno, Kimiyasu Shiraki, Masahiko Kurokawa

**Affiliations:** ^1^Department of Biochemistry, School of Pharmaceutical Sciences, Kyushu University of Health and Welfare, 1714-1 Yoshino, Nobeoka, Miyazaki 882-8508, Japan; ^2^Department of Pharmaceutical Health Sciences, School of Pharmaceutical Sciences, Kyushu University of Health and Welfare, 1714-1 Yoshino, Nobeoka, Miyazaki 882-8508, Japan; ^3^Department of Microbiology, School of Pharmaceutical Sciences, Kyushu University of Health and Welfare, 1714-1 Yoshino, Nobeoka, Miyazaki 882-8508, Japan; ^4^Amazonfood Ltd., 2-24-28 Ohhara, Setagaya, Tokyo 156-0041, Japan; ^5^Department of Food Science, College of Food Engineering, State University of Campinas, P.O. Box 6177, 13083-970 Campinas, SP, Brazil; ^6^School of Pharmacy, Nihon University, 7-7-1 Narashinodai, Funabashi-shi, Chiba 274-8555, Japan; ^7^Department of Virology, University of Toyama, 2630 Sugitani, Toyama 930-0194, Japan

## Abstract

Ethanol extracts (AF-06, 07, and 08, 10 mg/kg) of Brazilian propolis were administered orally to cutaneously herpes simplex virus type 1 (HSV-1)-infected mice three times daily on days 0 to 6 after infection to evaluate their efficacies against HSV-1 infection and significantly limited development of herpetic skin lesions. AF-07 and 08 significantly reduced virus titers in brain and/or skin on day 4 without toxicity, but AF-08 had no anti-HSV-1 activity *in vitro*. AF-06 and 08 significantly enhanced delayed-type hypersensitivity (DTH) to inactivated HSV-1 antigen in infected mice. Oral AF-08-administration significantly augmented interferon (IFN)-**γ** production by HSV-1 antigen from splenocytes of HSV-1-infected mice, while direct exposure of splenocytes of infected mice to AF-06 significantly elevated IFN-**γ** production *in vitro*. Thus, AF-08 might have components that are active *in vivo* even after oral administration and those of AF-06 might be active only *in vitro*. Because DTH is a major host defense for intradermal HSV-1 infection, augmentation of DTH response by AF-06 or 08, directly or indirectly, respectively, may contribute to their efficacies against HSV-1 infection. In addition, AF-06 and 07 possibly contain anti-HSV-1 components contributing to their efficacies. Such biological activities of Brazilian propolis may be useful to analyze its pharmacological actions.

## 1. Introduction

Propolis is currently used as an alternative medicine in the management of various ailments [1]. It has been used worldwide as a folk medicine since ca. 300 BC and as a dietary supplement to maintain or improve human health [2–4]. Propolis is a resinous hive product and consists of a mixture of plant exudates collected by honeybees and beeswax. The chemical composition of propolis is complex and varies with the area where the propolis was harvested because of the differences in plants and honeybees. Propolis has been reported to exhibit various biological activities, such as antibacterial, antitumor, and immunostimulatory activities [1, 2, 5–9]. It has been also shown to exhibit anti-influenza virus activity *in vitro* [6, 10] and antiherpes simplex virus (HSV) activity *in vitro* [11, 12]. Recently, Brazilian propolis was reported to possess anti-influenza virus activity and to ameliorate influenza symptoms in mice [13]. Although the efficacy of a Canadian propolis ointment against genital herpetic lesions caused by HSV type 2 in humans has been reported [14], the anti-HSV-1 activity of propolis *in vivo* is not clear. 

We have been studying the antiviral activity of medicinal herb extracts for their possible use in the management of viral infection in humans. In a series of studies, we have used a cutaneous HSV type 1 (HSV-1) infection model in mice because it manifests various markers in the development of the disease and because this model has been shown to have advantages for monitoring the efficacies of natural products such as herbal medicines [15–20]. Using this murine cutaneous infection model, we have previously found medicinal herbs that exhibit anti-HSV-1 activity *in vitro* and *in vivo *[15–20]. Kakkon-to, a kampo formulation composed of seven herbs, was shown to exhibit therapeutic anti-HSV-1 efficacy in the murine model by enhancing delayed-type hypersensitivity (DTH) response against HSV-1 antigen as assessed by cutaneous reaction [15]. The DTH response has been reported to be a major defense system in intradermal HSV-1 infection [21–23]. Thus, the DTH reaction is a useful immunological marker to evaluate the efficacy on intradermal HSV infection.

In this study, three kinds of Brazilian propolis harvested in different areas of Brazil were examined for their anti-HSV-1 efficacies in a cutaneous HSV-1 infection model in mice. We found that three ethanol extracts of propolis used in this study moderately alleviated the symptoms of cutaneous herpetic infection. The modes of their efficacies were evaluated virologically and immunologically, and we characterized the potential and pharmacological activities of Brazilian propolis.

## 2. Materials and Methods

### 2.1. Viruses and Cells

HSV-1 7401H strain [24] was propagated in Vero E6 cells [16]. The infected cultures were frozen and thawed three times and centrifuged at 3,000 rpm for 15 min. Their supernatants were stored at −80°C until use [16]. Vero E6 cells were grown and maintained in Eagle's minimal essential medium (MEM) supplemented with 5% and 2% heat-inactivated calf serum, respectively. Splenocytes were cultured in RPMI-1640 medium supplemented with 10% heat-inactivated fetal calf serum (FCS).

### 2.2. Propolis and Acyclovir (ACV)

Propolis was harvested in the different areas of Brazil, and the major botanical origin of propolis was estimated by our field-work as shown in Table 1. The voucher specimens AF0305 (AF-05), AF0306 (AF-06), AF0307 (AF-07), and AF0308 (AF-08) were deposited at Amazonfood Co., Ltd., Tokyo, Japan. The harvested propolis was extracted once with 95% ethanol (1 : 1, w/w) for 3 months at room temperature, and the ethanol-extracts were dried in a drying machine with vacuum at 37 to 38°C for 40 to 60 min. Finally propolis paste as ethanol extracts contained water at 5–7% (w/w), and the recovered yields were about 30% (w/w) of original propolis. The ethanol extracts, AF-05, 06, 07, and 08, were supplied by Amazonfood Co., Ltd., Tokyo, Japan. These extracts were dissolved in an appropriate volume of dimethyl sulfoxide (DMSO) and diluted with culture medium to make various final concentrations for *in vitro* assays. The concentration of DMSO in each medium was less than 0.2%. For *in vivo* assays, these extracts were dissolved in 1% DMSO and administered orally to mice. ACV was purchased from Sigma-Aldrich, Japan and dissolved in distilled water.

### 2.3. HPLC Analysis

AF-05, 06, 07, and 08 were dissolved in acetonitrile at 20 mg/mL, and 1 *μ*L was analyzed using coupling a reverse-phase high-performance liquid chromatograph (HPLC) system (LC-10ADvp, Shimadzu, Kyoto, Japan) to a diode array detector (SPD-M10Avp, Shimadzu, Kyoto, Japan). Separation was performed on a reverse-phase column (COSMOSIL 5C18-MS-II, 4.6 mm I.D. × 150 mm, 5 *μ*m; Nacalai, Kyoto, Japan) at 40°C using a mobile phase of water and acetonitrile. The elution was carried out with a nonlinear gradient of 20% acetonitrile for 10 min, a linear gradient of 20% to 100% of acetonitrile for 10 to 40 min, a nonlinear gradient of 100% of acetonitrile for 40 to 50 min, and a nonlinear gradient of 20% acetonitrile for 60 to 80 min at a flow rate of 0.7 mL/min. Spectrophotometric detection was conducted at 254 nm. Quercetin (Wako, Osaka, Japan), apigenin (Tokyo Chemical Industry, Tokyo, Japan), kaempferol (Tokyo Chemical Industry), chrysin (Tokyo Chemical Industry, Tokyo, Japan), acacetin (ChromaDex, Irvine, USA), and artepillin C (Wako) were used as authentic standards, and the major peaks eluted by HPLC were characterized by the analysis of ultraviolet absorbance at 200 to 400 nm.

### 2.4. Mice

BALB/c female (6 weeks old, 18–21 g) mice were purchased from Sankyo Labo Service Co., Ltd., Tokyo, Japan, or Kyudo Animal Laboratory, Kumamoto, Japan. The mice were housed five per cage in specific pathogen-free conditions, with food and water *ad libitum* and under a 12 h light/12 h dark diurnal cycle (light at 7.00 a.m.). The temperature in the room was kept at 24 ± 2°C. The mice were acclimated for at least five days before starting experimental procedures. Animal studies followed the animal experimentation guidelines of Toyama University and Kyushu University of Health and Welfare and were carried out in an approved biosafety level facility.

### 2.5. Murine HSV-1 Infection

The right midflank of each mouse was clipped and depilated with a chemical depilatory, Hair Remover (Shiseido, Co., Ltd., Tokyo, Japan). One or two days later, the naked skin was scratched using a 27-gauge needle and 5 *μ*L of HSV-1 (7401H strain) suspension containing 2 × 10^5^ or 1 × 10^6^ PFU was applied to the scarified area [18, 19]. Propolis extracts (10 mg/kg) or ACV (5 mg/kg) was orally administered in a volume of 0.25 mL/mouse to the mice by gavage, once 4 h prior to and twice after virus infection on day 0, and 3 times daily from day 1 to day 6 after infection. A 1% DMSO solution was used as a control. The dosage of 30 mg/kg/day of extracts for mice corresponds to about 12 times the dosage for humans based on body surface area [25] and used as a conventional dose of propolis [13]. Acyclovir at 5 mg/kg for mice has been shown to be significantly effective against HSV-1 infection in a murine infection model [18, 19]. Thus, we used the dose of 5 mg/kg for mice as a clinical dose. Ten mice in each group were weighed daily after infection, and the changes were calculated based on the body weight of each mouse on day 0. The development of skin lesions and mortality were continuously monitored every 8 h daily and scored as follows: 0, no lesion; 2, vesicles in local region; 4, erosion and/or ulceration in local region; 6, mild zosteriform lesion; 8, moderate zosteriform lesion; 10, severe zosteriform lesion; and 12, death. [16]. To assess the toxicity of extracts, four mice in each uninfected group were administered the extracts at 10 mg/kg following the same schedule used in infected mice. The uninfected mice were weighed daily as described above.

### 2.6. Determination of Virus Yields in Skin and Brain

Virus yields in the skin and brain were determined in infected mice. Mice were cutaneously infected with HSV-1 (2 × 10^5^ PFU/mouse), and propolis extracts were orally administered at doses of 10 mg/kg following the same schedule as described above. The brain and skin (whole lesions that include the area (5 × 5 mm) encompassing the inoculation site) were removed under anesthesia on day 4 after infection and homogenized in 2 mL of phosphate-buffered saline as described previously [18]. The homogenate was centrifuged at 3,000 rpm for 15 min, and the virus yield in the supernatant was determined by the plaque assay on Vero cells [16].

### 2.7. Plaque Reduction Assay and Cytotoxicity Assay

Duplicate cultures of Vero cells in 60 mm plastic dishes were infected with 100 plaque forming units (PFU)/0.2 mL of HSV-1 for 1 h. Then the cells were overlaid with 5 mL of nutrient methylcellulose (0.8%) medium containing various concentrations of propolis extracts. The infected cells were fixed and stained, and the number of plaques was counted [18]. The effective concentrations for 50% plaque reduction (EC_50_) were determined from a curve relating the plaque number to the concentration of samples [18].

 Cytotoxicity was examined by the growth inhibition of Vero cells as described previously [18]. Briefly, Vero cells were seeded at a concentration of 2.5 × 10^4^ cells/well in 24-well plates and grown at 37°C for 2 days. The culture medium was replaced with fresh medium containing extracts at various concentrations. Cells were further grown for 2 days. The cells were treated with trypsin, and the number of viable cells was determined by the trypan blue-exclusion test. Cytotoxic concentrations for 50% growth reduction (CC_50_) were determined from a curve relating percent cell viability to the concentration of drugs [18].

### 2.8. Footpad Swelling due to HSV-1 Antigen in Mice

To evaluate the effects of propolis extracts on the DTH reaction, we examined the skin reaction against HSV-1 antigen in mice infected with 7401H HSV-1 strain. The mice were intradermally infected with 10 *μ*L (7 × 10^5^ PFU/mouse) of 7401H strain using a microsyringe with a 27-gauge needle under anesthesia [15]. The propolis extracts were administered to the infected mice for day 0 to day 4 following the same schedule as described above. The infected mice were challenged by injection of 10 *μ*L containing 7 × 10^5^ PFU of ultraviolet light- (UV-) inactivated virus antigen into a footpad on day 4 after infection. The swelling of footpads was measured at 24, 36, and 48 h after the footpad challenge by using an engineer's micrometer, because DTH reaction in intradermally HSV-1-infected mice has been reported to be detected between 24 and 48 h after challenge [15, 21].

### 2.9. Interferon (IFN)-*γ* Production from Splenocytes

Effects of the oral administration of extracts on IFN-*γ* production from splenocytes of HSV-1-infected mice were examined. Intradermally HSV-1-infected mice were orally administered propolis extracts as described above. On day 4 after infection, spleens were removed under anesthesia and splenocytes were prepared after erythrocytolysis. The splenocytes prepared from three mice in a group were combined, and the mixed splenocytes were incubated at 6 × 10^5^ cells/well in 96-well plates, in octuplicate, in RPMI-1640 medium supplemented with 10% heat-inactivated FCS in the presence or absence of UV-inactivated HSV-1 antigen at 2 × 10^4^ PFU/well for 24 h at 37°C. Cell supernatants of the cultures in the 96-well plates were harvested after centrifugation and stored at −80°C for ELISA of IFN-*γ*.

In addition, to examine the direct effect of propolis extracts on the IFN-*γ* production from splenocytes of HSV-1-infected mice, splenocytes were prepared on day 4 after infection from intradermally HSV-1-infected mice without oral administration of the extracts. The splenocytes were cultured in the presence of 0, 0.01, 0.03, 0.1, 0.3, 1, 3, and 10 *μ*g/mL of propolis extracts and in the presence or absence of HSV-1 antigen as described above.

### 2.10. ELISA

IFN-*γ* levels in the culture supernatants were measured using a specific ELISA kit (Ready-set-go, eBioscience Inc., San Diego, CA) according to the manufacturer's instructions. The product was tested and found to conform to all eBioscience Inc. Quality Control release specifications. The lower limit of detection sensitivity of the kit is 15 pg/mL. The intra- and interassay coefficients of variation for these ELISA were less than 10%.

### 2.11. Statistical Analysis

Statistical significances of differences between the EC_50_ and CC_50_ values and in the changes of net body weights of infected mice, virus titers, net increase in footpad thicknesses, and IFN-*γ* levels were evaluated using Student's *t*-test. Interactions between mean scores of treated and untreated groups were analyzed using the repeated measures two-way ANOVA. A *P* value of  .05 or less was considered to be significant statistically.

## 3. Results

### 3.1. Compositions of Propolis Extracts by HPLC

**Figure 1:**
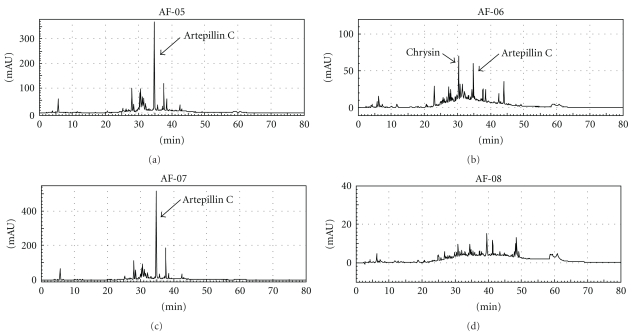
HPLC profiles of AF-05, 06, 07, and 08.

Compositions of four propolis extracts (AF-05, 06, 07, and 08) harvested in different areas of Brazil were analyzed by HPLC. As shown in Figure 1, the HPLC patterns of AF-05 and AF-07 were similar and the patterns of AF-06 and AF-08 were obviously different from those of AF-05 and 07. As the major peaks of our HPLC profiles were estimated as flavonoids or prenylated phenylpropanoids in comparison with those of Brazilian propolis as reported previously by Park et al. [26], we detected some of them in our HPLC system. Quercetin, apigenin, kaempferol, chrysin, acacetin, and artepillin C as authentic compounds were eluted at 21.8, 24.6, 25.1, 29.8, 29.9, and 34.4 min, respectively. The highest peak in AF-05 and AF-07 and the second higher peak in AF-06 were characterized as artepillin C by their analyses of ultraviolet absorptions, and the highest peak in AF-06 was done as chrysin. As compared with AF-05, 06, and 07, artepillin C and chrysin were not major in AF-08. We selected AF-06, 07, and 08 as propolis with different compositions.

### 3.2. Efficacies of Propolis Extracts on Murine HSV-1 Infection Model

**Figure 2:**
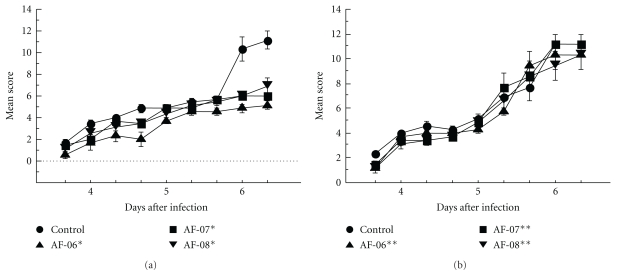
Effects of propolis ethanol extracts on the development of herpetic skin lesions of mice infected with HSV-1 cutaneously. Ten mice in each group were infected with HSV-1 at 2 × 10^5^ (a) or 1 × 10^6^ (b) PFU/mouse and administered water (●), AF-06 (▲), AF-07 (■), or AF-08 (▾) as described in text. *P < .003 and **P < .05 versus control by repeated measures ANOVA.

The efficacies of AF-06, 07, and 08 were examined in a cutaneous HSV-1 infection model in mice (Figure 2). Previously, AF-08 at 10 mg/kg was significantly effective in prolonging the survival times of influenza virus-infected mice and in reducing virus yields in the bronchoalveolar lavage fluids of lungs in infected mice [13]. Therefore, all three propolis extracts (AF-06, 07, and 08) were administered at 10 mg/kg as a biologically active dose. In mice infected with HSV-1 at 2 × 10^5^ PFU/mouse, all three were moderately but significantly effective in limiting the development of skin lesions for days 3.3 to 6.3 after infection (Figure 2(a), *P* < .003 by the repeated measure ANOVA), although the mortality under propolis administration was similar to that of the control (water-administered mice) and all infected mice in each group examined were dead until 9 days after infection. There was no significant difference between the changes of net body weights of mock-infected mice administered water and AF-06, 07, or 08 on the tenth day after infection (2.6 ± 0.5 and 2.7 ± 0.4, 2.5 ± 0.3, or 2.6 ± 0.2 g, resp.). When mice were infected with the higher titer of 1 × 10^6^ PFU/mouse, the period of significant limitation of skin lesion development was shortened, but the development was still significantly limited for days 3.3 to 5.0 (Figure 2(b), *P* < .05 by the repeated measure ANOVA). It was confirmed that all three propolis extracts moderately delayed the development and progression of herpetic skin lesions in an early phase of infection without toxicity.

### 3.3. Anti-HSV-1 Activity of Propolis Extracts in Mice and In Vitro

To evaluate the potential anti-HSV-1 activity of the three propolis extracts in mice, effects of the extracts on virus titers in the skin and brain of infected mice were examined on day 4 after infection, as shown in Table 1. AF-07 and ACV were significantly effective in reducing virus titers in the skin and brain of infected mice compared with the control (*P* < .05 or *P* < .001 by Student's *t*-test), and AF-08 was effective in reducing virus titer in only the brain (*P* < .001 by Student's *t*-test). However, AF-06 had no significant effect on virus titers in either skin or brain. 

To assess the potential anti-HSV-1 activity of the three propolis extracts *in vitro*, we performed a plaque reduction assay in Vero cells. As shown in Table 2, the EC_50_ values of AF-06 and 07 were moderately but significantly lower than the CC_50_ values although the selectivity indexes (CC_50_/EC_50_) of AF-06 and 07 were very lower than that of ACV. However, the selectivity index of AF-08 was less than 0.9. Thus, it is possible that AF-06 and 07 possess potential anti-HSV-1 activity *in vitro*.

### 3.4. Effect of Propolis Extracts on DTH Reaction to HSV-1 Antigen

**Figure 3:**
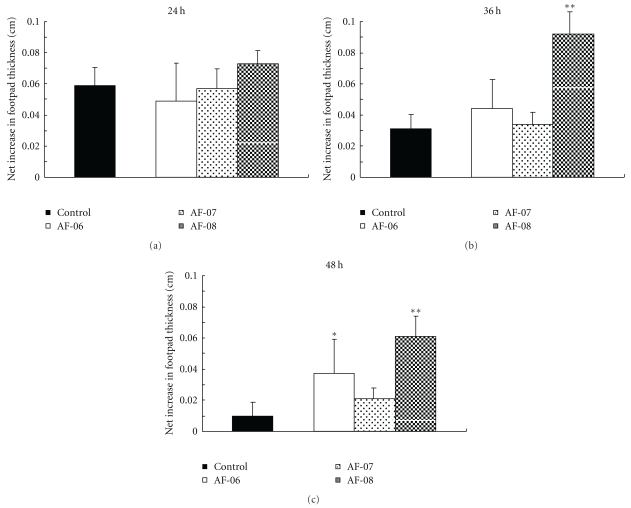
Time course of skin reactions to HSV-1 antigen in mice infected intradermally with HSV-1. Seven HSV-1-infected mice in a group were administered water (closed columns), AF-06 (opened columns), AF-07 (lightly dotted columns), or AF-08 (densely dotted columns), and the mean percent swelling was determined at 24, 36, and 48 h after injection of HSV antigen into footpads as described in text. *P < .05 and **P < .01 versus control by Student's *t*-test. Bars indicate SEM.

To evaluate the immunological effect of the three propolis extracts in mice infected with HSV-1, effects of the extracts on DTH reaction, which is one of the most important defense systems in intradermal HSV-1 infection, were examined [21–23]. As shown in Figure 3, AF-06 and 08 augmented the DTH skin reaction, a major immune defense response to intradermal HSV infection, at 36 h and/or 48 h after challenge of HSV-1 antigen but AF-07 did not.

### 3.5. Effects of Propolis Extracts on IFN-*γ* Production by Splenocytes of Infected Mice

IFN-*γ* is a major mediator of lymphocyte recruitment in DTH [27]. Oral administration of AF-06, 07, and 08 was examined for their effects on IFN-*γ* production by HSV-1 antigen from splenocytes prepared from mice infected intradermally with HSV-1. As shown in Table 3, the oral administration of AF-08 was significantly effective in augmenting IFN-*γ* production by HSV-1 antigen from the splenocytes, but AF-06 and 07 were not. In infected mice administered AF-08, the significant increase of IFN-*γ* production correlated with augmentation of the DTH skin reaction but did not in infected mice administered AF-06 and 07. 

**Figure 4:**
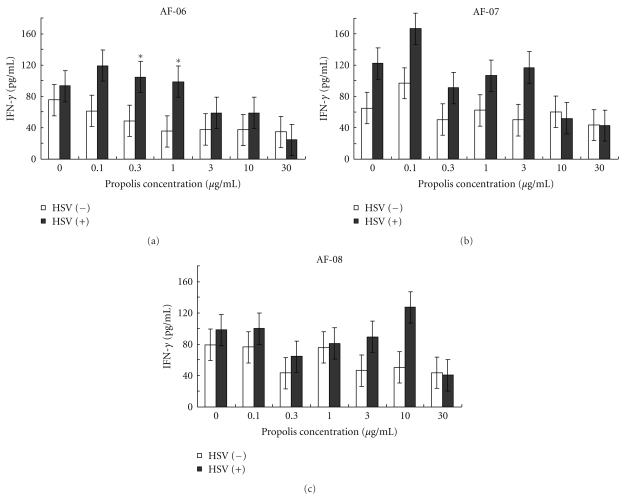
Effects of AF-06, 07, and 08 on IFN-*γ* production from splenocytes by inactivated HSV antigen (Table 4). Splenocytes were prepared from intradermally HSV-1-infected mice on day 4 and incubated in the presence of various concentrations of extracts (0, 0.1, 0.3, 1, 3, 10, and 30 *μ*g/mL) and in the presence (closed columns) or absence (opened columns) of HSV antigen for 24 h. IFN-*γ* levels in the culture supernatants were measured by ELISA as described in text. *P < .05 versus HSV(−) by Student's *t*-test. Bars indicate SEM.

 On the other hand, IFN-*γ* production from splenocytes prepared from HSV-1-infected mice without administration of propolis extracts was examined in the presence of various concentrations of the extracts with or without inactivated HSV-1 antigen. As shown in Figure 4, AF-06 at 0.3 and 1 *μ*g/mL significantly augmented IFN-*γ* production in the presence of inactivated HSV-1 antigen as compared with that in its absence. AF-06 exhibited the *bell *-*shaped concentration* dependency that is a characteristic of cytokine reaction by its direct stimulation to splenocytes. However, AF-07 and 08 failed to augment the production in the presence of the antigen at all concentrations examined.

## 4. Discussion

Propolis is a natural product known worldwide as a folk medicine and used as a dietary supplement because of its wide range of biological activities [1–9]. It has been administered orally and applied for the improvement of symptoms of viral infection. However, there has been little research to elucidate the theoretical bases for the clinical efficacy of propolis. In this study, we tried to determine the basis of the therapeutic effects on herpetic skin lesions of propolis using a cutaneous HSV-1 infection mouse model and found that the ethanol extracts of Brazilian propolis AF-06, 07, and 08 exhibited therapeutically moderate efficacy in limiting herpetic lesions in mice. Brazilian propolis has been reported to have a chemical composition and pharmacological activity that are different from propolis produced in temperate zones [3, 7–9, 28]. Even among Brazilian propolis, the chemical composition depends on the area and vegetation where it was harvested. Expectedly the HPLC patterns of compositions of AF-05 and 07, in which artepillin C is a major component, were similar, because they were harvested in similar area and vegetation as shown in Table 1. This result was consistent with that AF-05 and 07 physicochemically belong to a same group that is different from the groups of AF-06 and 08 [8, 26]. AF-06 and 08 were harvested in similar area but their vegetations (Table 1) and HPLC patterns (Figure 1) were different. Although we selected three propolis extracts (AF-06, 07, and 08) that have different compositions, all three were effective against cutaneous HSV-1 infection in mice. Thus, Brazilian propolis may be characterized as having possible antiherpes components* in vivo*.

 The three different propolis extracts moderately limited herpetic skin lesions in the early phase of cutaneous HSV-1 infection (Figure 2). We used 10 mg/kg of extracts for oral administration to mice. This dose for mice corresponds to the conventional dose of propolis used for humans based on body surface area. The virus titers in skin and brain of infected mice administered AF-07 and 08 at this dose were equivalent to that of 5 mg/kg oral ACV treatment, to the clinical dose for humans (Table 1). It suggested the possibility that oral administration of AF-07 and 08 could be used clinically in combination with oral ACV treatment in the early phase of cutaneous HSV-1 infection to improve the efficacy of ACV. 

Among AF-06, 07, and 08, AF-07 and 08 were moderately effective in reducing virus titers in the skin and/or brain of infected mice (Table 1). AF-07 and 08 were possibly effective in producing anti-HSV-1 activity in mice. AF-07 showed modest anti-HSV-1 activity *in vitro* (Table 2), and the anti-HSV-1 activity of AF-07 was also suggested to be maintained in the skin and brain of infected mice after oral administration. As the composition of AF-05 was similar to that of AF-07 (Figure 1), it is possible that AF-05 also exhibits anti-HSV-1 activity in mice. In contrast with AF-07, AF-08 did not exhibit anti-HSV-1 activity *in vitro* (Table 2) but significantly reduced virus titers in the brain of infected mice (Table 1). It is possible that AF-08 has an immunological activity contributing to the limitation of HSV-1 growth and/or elimination of viruses in the brain. In the case of AF-06, it was significantly effective against HSV-1 *in vitro* (Table 2) but not *in vivo* (Table 1). The anti-HSV-1 activity of AF-06 exhibited *in vitro* was probably noneffective in mice after oral administration. However, AF-06 was effective in limiting the development of herpetic skin lesions (Figure 2). AF-06 was suggested to have an immunological activity associated with the limitation of herpetic skin lesions. Thus all three propolis extracts used were suggested to have some components showing anti-HSV-1 activity or immunological activity leading the alleviation of herpetic symptoms. 

The DTH skin reaction has been shown to be a major defense system in the clearance of HSV-1 in mice infected intradermally with HSV-1 [21, 23], although recovery from intraperitoneal HSV-1 infection has been shown to be mediated by cytotoxic T lymphocytes [22]. The oral administration of AF-06 and 08 significantly augmented DTH skin reactions against inactivated HSV-1 antigen but that of AF-07 did not (Figure 3). In intradermally HSV-1-infected mice, DTH reaction has been reported to be maximum between 24 and 48 h after challenge [15, 21]. The significant augmentation of swelling at 36 and 48 h after challenge by AF-06 and 08 (Figure 3) was consistent with the previous reports. Thus, AF-06 and 08 were suggested to have components that act as biological response modifiers (BRMs) for DTH reaction in intradermally HSV-1-infected mice. In contrast to AF-06 and 08, AF-07 exhibited anti-HSV-1 activity *in vitro* and *in vivo* (Tables 1 and 2) and probably contains components that exhibit anti-HSV-1 activity rather than act as BRM. 

IFN-*γ* has been reported to be a potent stimulator of lymphocyte migration into skin and a major mediator of lymphocyte recruitment in DTH [27]. The oral administration of AF-08 was significantly effective in elevating HSV-1 antigen-stimulated IFN-*γ* production from the splenocytes of HSV-1-infected mice (Table 3). However, AF-08 failed to directly augment the IFN-*γ* production from splenocytes of HSV-1-infected mice in the presence of HSV-1 antigen (Figure 4). Thus, AF-08 was suggested to have some components that can be active as BRM in infected mice after oral administration. On the other hand, AF-06 was not significantly effective in augmenting IFN-*γ* production in infected mice after oral administration (Table 3) but directly augmented IFN-*γ* production from splenocytes *in vitro* (Figure 4). This indicated that AF-06 has components that can act directly on splenocytes as BRM *in vitro*. However, because AF-07 was not effective in the DTH skin reaction and IFN-*γ* production from splenocytes by HSV-1 antigen, its efficacy was confirmed to be possibly due to anti-HSV-1 activity of the components. The DTH reaction against HSV-1 antigen was reported to be mainly mediated by Lyt 1^+^2^−^ T-lymphocyte subsets in an intradermal infection model [29, 30]. Macrophages activated by the T-lymphocytes may be responsible for the DTH reaction [31]. Components of AF-06 and 08 might have activated Lyt 1^+^2^−^ T-lymphocyte subsets or directly activated macrophages in the site of skin lesions of infected mice. AF-06 and 08 were suggested to have components that act as BRM. 

Brazilian propolis contains various kinds of flavonoids and prenylated phenylpropanoids [8, 26]. In this study, artepillin C as a prenylated phenylpropanoid was a major component in AF-05, 06, and 07 and chrysin as a flavonoid was a major component in AF-06 (Figure 1). These results suggested that major components of Brazilian propolis are flavonoids and prenylated phenylpropanoids and supported other works with Brazilian propolis [8, 26]. Brazilian propolis is also known to have the wide range of biological activities [2–5, 7, 9]. It may be worthwhile to further investigate anti-HSV-1 or immunologically active compounds from flavonoids and prenylated phenylpropanoids that may be involved in AF-05, 06, 07, and 08 used in this study.

 We characterized the experimental bases of Brazilian propolis treatment using an HSV-1 infection model in mice. Together, all results indicate that Brazilian propolis showed not only direct anti-HSV-1 activity but also immunological activity against intradermal HSV-1 infection in mice. Especially, the immunological activity associated with IFN-*γ* production-inducing Th1 immunity in mice may contribute to the elucidation of various pharmacological actions of propolis in health and disease.

## Figures and Tables

**Table 1:** Features of propolis.

Propolis	Species*	Family*	State**
AF-05	*Baccharis dracunculifolia* DC	Compositae	Minas Gerais
AF-06	*Baccharis erioclada* DC	Compositae	Rio Grande do Sul
AF-07	*Baccharis dracunculifolia* DC	Compositae	Minas Gerais
AF-08	*Myrceugenia euosma* (Berg) D. Legrand	Myrtaceae	Rio Grande do Sul

*Major botanical origins in areas where propolis was collected.

** Brazilian states where propolis was collected.

**Table 2:** Effect of propolis extracts on virus titers in skin and brain of HSV-1-infected mice.

Administration	Virus titer (log_10_ PFU/organ)	
	Skin	Brain
Control	6.1 ± 0.07	2.2 ± 0.02
AF-06	6.3 ± 0.11	1.6 ± 0.48
AF-07	5.7 ± 0.09*	<1.0 ± 0.00**
AF-08	6.1 ± 0.04	<1.0 ± 0.00**
ACV (5 mg/kg)	5.4 ± 0.14*	<1.0 ± 0.00**

Values are mean ± standard error of the mean (SEM) of five mice.

*P < .01 and **P < .001 versus control by Student's *t*-test.

**Table 3:** Anti-HSV-1 activity of propolis extracts in Vero cells.

	EC_50 _(*μ*g/mL)	CC_50 _(*μ*g/mL)	CC_50_/EC_50_
AF-06	61.0 ± 0.2*	93.9 ± 1.8	1.5
AF-07	51.4 ± 1.3*	82.0 ± 0.3	1.6
AF-08	>50	43.4 ± 2.6	<0.9
ACV	0.48 ± 0.05	>30	>62.5

Values are mean ± SEM of three independent experiments.

*P < .05 versus CC_50_ by Student's *t*-test.

**Table 4:** IFN-*γ* production by inactivated HSV antigens from splenocytes of HSV-1-infected mice treated orally with propolis extracts.

	HSV− (pg/mL)	HSV+ (pg/mL)
Control	<15	<15
AF-06	<15	17.47 ± 8.18
AF-07	<15	<15
AF-08	<15	52.12 ± 15.87*

Values are mean ± SEM of octuplicate wells prepared from splenocytes of three mice.

*P < .005 versus control by Student's *t*-test.
